# A modular platform to generate functional sympathetic neuron-innervated heart assembloids

**DOI:** 10.21203/rs.3.rs-3894397/v1

**Published:** 2024-03-21

**Authors:** Nadja Zeltner, Hsueh-Fu Wu, Kenyi Saito-Diaz, Xin Sun, Ming Song, Tripti Saini, Courtney Grant, Christina James, Kimata Thomas, Yohannes Abate, Elizabeth Howerth, Peter Kner, Bingqian Xu

**Affiliations:** Universtiy of Georgia; Universtiy of Georgia; Universtiy of Georgia; Universtiy of Georgia; Universtiy of Georgia; Universtiy of Georgia; Universtiy of Georgia; Universtiy of Georgia; Universtiy of Georgia; University of Georgia, Athens, Georgia 30602, USA; Universtiy of Georgia; Universtiy of Georgia; University of Georgia

## Abstract

The technology of human pluripotent stem cell (hPSC)-based 3D organoid/assembloid cultures has become a powerful tool for the study of human embryonic development, disease modeling and drug discovery in recent years. The autonomic sympathetic nervous system innervates and regulates almost all organs in the body, including the heart. Yet, most reported organoids to date are not innervated, thus lacking proper neural regulation, and hindering reciprocal tissue maturation. Here, we developed a simple and versatile sympathetic neuron (symN)-innervated cardiac assembloid without the need for bioengineering. Our human sympathetic cardiac assembloids (hSCAs) showed mature muscle structures, atrial to ventricular patterning, and spontaneous beating. hSCA-innervating symNs displayed neurotransmitter synthesis and functional regulation of the cardiac beating rate, which could be manipulated pharmacologically or optogenetically. We modeled symN-mediated cardiac development and myocardial infarction. This hSCAs provides a tool for future neurocardiotoxicity screening approaches and is highly versatile and modular, where the types of neuron (symN or parasympathetic or sensory neuron) and organoid (heart, lung, kidney) to be innervated may be interchanged.

## Introduction

Human pluripotent stem cell (hPSC)-based 3D organoid strategies have become powerful tools to study human organ development, for disease modeling and drug discovery over the past years. Assembloids result from the integration of multiple organoids or a combination of organoids with other cell types^1–3^. Organoids of various organs have been described, including brain, kidney, heart, lung, liver and more^4–8^. Most organs of the body, except the brain, are innervated and thus regulated by the autonomic nervous system. Specifically, the sympathetic nervous system is responsible for activation of cells in most organs. A key challenge in the organoid field is the lack of innervation of organoids, despite neural regulation being vital for organ and thus organoid development, integrity, and function. To address this, we present a simple, easy to reproduce and cost-effective method without the need for bioengineering or special instrumentation to create sympathetic neuron (symN)-innervated cardiac organoids. The modular approach allows adaptation for innervation of various organoid types, like lung, kidney, or liver, by using symNs; or for innervation of cardiac organoids by other neuron types, such as parasympathetic or sensory neurons.

Innervation of peripheral tissue organoids has been reported. Intestinal organoids were innervated via mixing of vagal neural crest cells into human intestinal organoids (HIOs)^9^. This elegant study resulted in organoids containing functional enteric neurons and glia. However, the enteric nervous system is specific to the gastrointestinal tract and thus cannot easily be adapted for innervation of other organ type organoids, thus it lacks versatility. Neuromesodermal progenitor (NMP)-derived organoids were reported, where the NMP gave rise to both skeletal muscle and motor neurons from the same progenitor within the same cultures^10^, as an one-pot differentiation^11^. This exciting study, however, is not easily adaptable to other organ type organoids, if a common neural-tissue specific progenitor is not readily available. For example, it would be challenging to differentiate symN-innervated lung organoids, since there are no common lung and symN progenitors known. Also, one-pot differentiation protocols make it difficult to perform cell type-specific, genetic manipulations within the organoids. Schneider et al., showed exciting results of bioengineered heart organoids with assembled autonomic organoids creating an innervated cardiac assembloid^12^. This elegant study requires advanced bioengineering capabilities and isometric force instrumentation for analysis^12^. Such approaches are not easily accessible to many researchers. We describe a simple, highly versatile strategy for sympathetic innervation of cardiac organoids, that may be adapted to most organ’s organoid types and to other neuron types by other researchers.

Cardiovascular disease affects millions of people worldwide, and costs billions to the health care systems^13^. The most well-known and life-threatening cardiovascular diseases, such as heart failure and heart attack, may happen when blood supply is not regulated properly^13^. In addition to genetic heart defects, risk factors for heart failure and heart attack include metabolic conditions, such as diabetes and obesity, hypertension, viral infection, drug abuse, smoking, and excessive alcohol intake^13^. Interestingly, many of these risk factors are mediated by the sympathetic nervous system (SNS). The SNS belongs to the autonomic nervous system and regulates various cardiovascular functions, including heart rate, blood pressure, blood glucose, and gland secretion^14^. SymN hyperactivity and neuropathy have been associated with the pathology of diabetes and obesity, hypertension, viral infection, drug abuse, smoking, and alcohol toxicity in adults^15–20^. Furthermore, symNs innervate the heart as early as the embryonic stage (about E13.5), which may participate in hyperplastic to hypertrophic transition of the heart at neonatal stage and cardiac maturation^21–23^. Current studies have shown that developmental disorders that lead to aberrant symN innervation to the heart will cause heart rate variability and arrythmia, which increase the risk of sudden cardiac death^24–27^.

Here, we developed a novel, easy to reproduce and highly versatile neurocardiac organoid system using the assembloid technology, which refers to the assembly of more than two pre-differentiated cell types into an organoid structure^1,2,28^. The assembloid strategy has recently become popular, since it enables (i) the modeling of complex organs, (ii) modeling of interactions between various organs, or (iii) modeling of different regions in the body (for example in the brain)^1,2,28^. Given that we have recently established a chemically-defined, well-characterized protocol to differentiate highly pure symNs from hPSC^29,30^, and that there are many well-established CMs or cardiac organoid differentiation protocols available^31,32^, we sought to build a sympathetic-cardiac organoid as an assembloid. Herein, we combined early symN progenitors with early cardiac progenitors to form assembloids, named human sympathetic cardiac assembloids (hSCAs). The process of hSCA assembly is in free floating 3D culture, which is relatively easy to work with and reproducible by many stem cell labs. Cardiac tissues in our hSCAs were innervated by symNs and showed structural and functional maturation. SymNs in the organoids were functional and were able to regulate cardiac contraction and maturation. Hypoxic stress induced endogenous NE over secretion, and synergistically led to cardiac infarction features in hSCAs. This symN-heart axis assembloid paradigm therefore provides not just an ideal platform for assessing the mechanisms that environmental factors exert on cardiac function through sympathetic regulation, but also a playbook for generating innervated organoids from other organ systems.

## Results

### Generation of hSCAs

**Figure 1:**
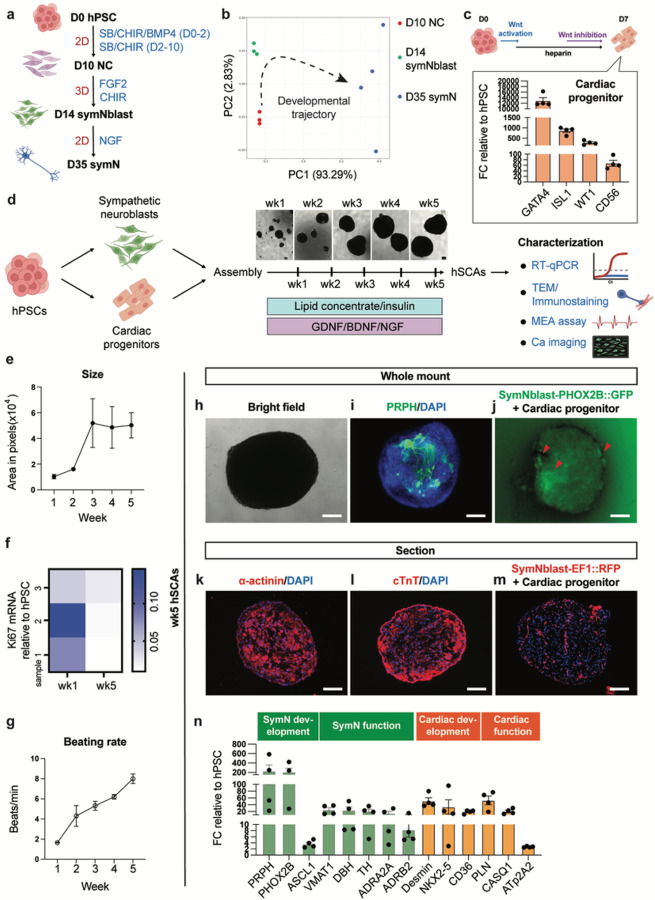
Assembly of hSCAs. (**a**) Schematic illustration of symNblast and symN differentiation from hPSCs. 2D: attached cell culture. 3D: suspending spheroid culture. (**b**) PCA plot showing the differentiation trajectory of symN differentiation. (**c**) Top: Schematic illustration of cardiac progenitor differentiation. Bottom: RT-qPCR analysis of day 7 cardiac progenitors. n=4 biological replicates. (**d**) Schematic illustration of hSCA assembly and differentiation. Representative right field images of hSCA growth among time. (**e**) Quantification of hSCA size overtime. n=3 biological replicates. (**f**) Heatmap of RT-qPCR analysis of wk1 and wk5 hSCAs for Ki67. n=3 biological replicates. (**g**) hSCA beating analysis overtime using the ImageJ Time Series Analyzer. n=6 biological replicates. (**h**) Representative bright field image of wk5 hSCAs. (**i**) Representative whole mount image of wk5 hSCAs for PRPH. (**j**) Representative whole mount image of wk5 hSCAs for PHOX2B::GFP reporter. (**k**) Representative cryosection image of wk5 hSCAs for α-actinin. (l) Representative cryosection image of wk5 hSCAs for cTnT. (**m**) Representative cryosection image of wk5 hSCAs for EF1::RFP reporter. (n) RT-qPCR analysis of wk5 hSCAs for symN and cardiac development markers. Error bars represent SEM. Scale bars represent 200 μm.

The sympathetic nervous system consists of two neural parts, the preganglionic and postganglionic symNs. While the preganglionic symNs are spinal motor neurons derived from the neuroectoderm, and therefore belong to the central nervous system, postganglionic symNs are the ones that innervate target tissues and communicate via the norepinephrine (NE) neurotransmitter^20^. We previously have established a directed differentiation protocol for postganglionic symNs (Fig. 1a)^29,30^. In this protocol, hPSCs are first differentiated into SOX10^+^ neural crest cells (NCCs), the progenitors of peripheral neurons^30^, in 2D, adherent cultures, with high efficiency (about 90%^30^). By replating the differentiated NCCs into 3D spheroids, the early sympathetic progenitor markers, *PHOX2B, ASCL1, HAND2, GATA2, GATA3*, are induced, representing sympathetic neuroblasts (symNblast)^29,30^. Neurons derived from symNblasts yield high purity (about 80%^29^) express typical symN markers, including nicotinic receptor CHRNA3/B4, adrenergic receptor ADRA2A/B2, vesicular monoamine transporter VMAT1/2, tyrosine hydroxylase TH, and norepinephrine transporter NET^29,30^. hPSC-derived symNs are spontaneously firing action potentials, which can be upregulated by nicotine treatment, and they can form connections to CMs in 2D co-cultures^29,30^. Bulk RNA seq. analysis (Wu et al., https://papers.ssrn.com/sol3/papers.cfm?abstract_id=4318816)^29^ showed that this protocol recapitulates the proper developmental stages, from neural crest to symN progenitors to symNs (Fig. 1b).

The heart consists of multiple cardiac lineages, including smooth muscle cells (of the vasculatures), cardiac epithelial and endothelial cells, and cardiac fibroblasts that produce the extracellular matrix^33,34^. For our hSCAs, we chose a CM differentiation protocol modified from Lin et al.,^35,36^ to generate early cardiac progenitors, which may still have the multipotency to be differentiated into several of the cardiac lineages^37,38^. hPSCs are cultured in 2D, and first induced by the WNT activator CHIR99021, followed by WNT inhibition using XAV939. Cardiac progenitors expressed typical markers, including *GATA4, WT1, ISLET-1*, and *CD56* shown by RTqPCR on day 7 (Fig. 1c). To form hSCAs, dissociated symNblasts (day 14) and cardiac progenitors (day 7) were mixed and cultured in low attachment plates, in floating cultures on a shaker for up to 5 weeks (Fig. 1d). The size of the hSCAs increased until week 3 and stabilized after (Fig. 1d–e), which was in line with decreased *Ki67* expression from wk1 to wk5 (Fig. 1f). The hSCAs were beating spontaneously with a beating rate that increased over time (Fig. 1g), which we quantified via video-based image quantification (**Suppl. Figure 1, movie S1**). These results suggest that on wk5, hSCAs have stopped proliferating and may have entered their maturation stage.

On wk5, we assessed the cellular composition in hSCAs by whole mount staining. Peripheral neural marker PRPH labeled neurons growing inside the organoids (Fig. 1i). By differentiating symNblast from a hPSC line that carries a PHOX2B-driven GFP reporter, we confirmed that the neurons in hSCAs are PHOX2B^+^ symNs (Fig. 1j). Upon cryosectioning of the hSCAs, we confirmed the presence of α-actinin^+^ and cTnT^+^ cardiac cells (Fig. 1k, l). Using an EF1-driven RFP reporter hPSC line, we differentiated symNblasts, assembled into hSCAs and showed that symNs are growing inside the cardiac mass, and are more abundant on the outskirts (Fig. 1m). Such a pattern of neural distribution is similar to the one seen in the living heart^21,39^. Accordingly, RT-qPCR analysis on wk5 hSCAs detected markers indicating both symN (*PRPH, PHOX2B, ASCL1*) and cardiac development (*PLN, Desmin, NKX2.5*), as well as maturation (*DBH, ADRA2A/B2, VMAT1, ATpA2, CD36, CASQ1*, Fig. 1n).

### hSCAs self-organization

**Figure 2:**
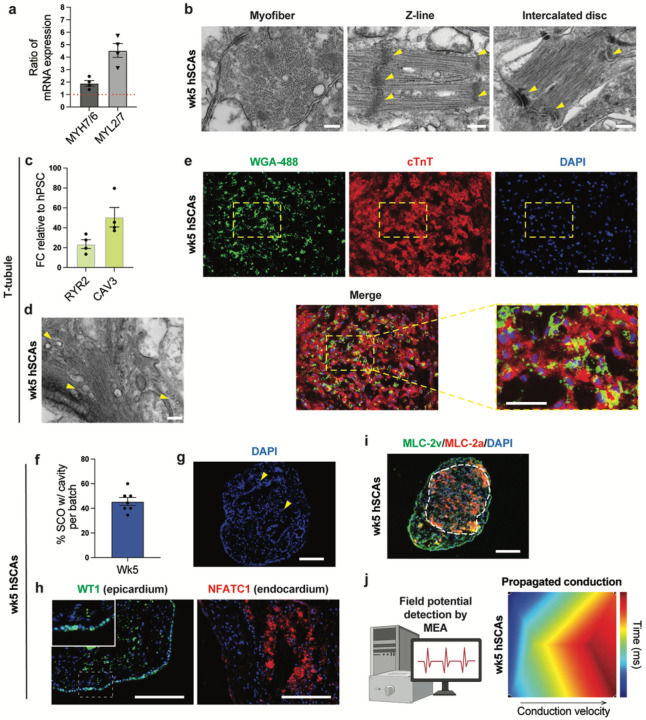
hSCAs are self-organized and display features of maturation. (**a**) RT-qPCR analysis of wk5 hSCAs for cardiac maturity markers. (**b**) Representative TEM images with yellow arrows indicating the myofiber, Z-line, and intercalated disc in hSCAs. Scale bars represent 200 nm. (**c**) RT-qPCR analysis of wk5 hSCAs for cardiac T-tubule markers. (**d**) Representative TEM images with yellow arrows indicating the T-tubules in hSCAs. Scale bar represents 200 nm. (**e**) Representative cryosection image of wk5 hSCAs which were stained for WGA-488 and cTnT to label T-tubules. Scale bars represent 200 μm, and 50 μm in the yellow dashed rectangle. (**f**) Quantification for the percentage of hSCAs with cavity structures. n=7 biological replicates. (**g**) Representative cryosection image of wk5 hSCAs with DAPI showing the cavity structures. Scale bar represents 200 μm. (**h**) Representative cryosection image of wk5 hSCAs for epicardial marker WT1 and endocardial marker NFATC1. Scale bars represent 200 μm. (**i**) Representative cryosection image of wk5 hSCAs for atrial marker MLC-2a and ventricular marker MLC-2v. Scale bar represents 200 μm. (**i**) Beating pattern of wk5 hSCAs was analyzed using MEA. Heatmap shows representative pattern of propagated conduction. Error bars represent SEM.

One of the gold standards for a qualified organoid/assembloid is its ability of cellular and structural self-organization, often indicating maturation and functionality^40^. Therefore, we first evaluated the maturity of the cardiac tissue within the hSCAs on wk5 via the ratio of MYH7/6 and MYL2/7 expression, which indicate human cardiac tissue maturity if they are above 1^41^. In wk5 hSCAs, both *MYH7* and *MYL2* levels were higher than *MYH6* and *MYL7*, respectively (Fig. 2a). To further examine the hSCAs maturity and organization, we assessed wk5 hSCAs by transmission electron microscopy (TEM). We observed the ultra-structures of well-aligned myofiber bundles, Z-lines, and intercalated discs (Fig. 2b). In addition, transverse tubules (t-tubules) are internalized CM membranes that surround the muscle fibers and are enriched in ion channels, which are crucial for mature excitation-contraction coupling and heart function^42,43^. RT-qPCR analysis of wk5 hSCAs identified the expressions of T-tubule markers RYR2 and *CAVEOLIN3* (Fig. 2c). Accordingly, we detected T-tubule protein structures within the cardiac tissues using TEM (Fig. 2d), and via wheat germ agglutinin (WGA) staining (Fig. 2e).

Interestingly, in about 50% of hSCAs in each differentiation (Fig. 2f), we observed cavity structures (Fig. 2g). Cavities were compartmentalized by a single layer of cells, excluding the possibility that the cavities are caused by necrosis that is often seen in long-term cultured organoids^44^. Similarly, a single cell layer was also formed in the exterior of wk5 hSCAs (Fig. 2g). To assess whether the cavity structures are mimicking heart chambers, we stained hSCAs for epicardium marker WT1 and endocardium marker NFATC1. The results showed that the exterior cell layer was WT1^+^, while the interior layer was TFATC1^+^ (Fig. 2h), suggesting both epicardium and endocardium patterning within the hSCAs. Furthermore, hSCAs showed polarized patterning for the atrial marker MLC-2a and ventricular marker MLC-2v (Fig. 2i). Using microelectrode array (MEA), we detected a propagating beating pattern in hSCAs (Fig. 2j). Together, our data suggest that wk5 hSCAs are self-organized in their structure and cell type variety, are relatively mature, and the tissues are functional.

### Functional coupling and sympathetic regulation in hSCAs

**Figure 3:**
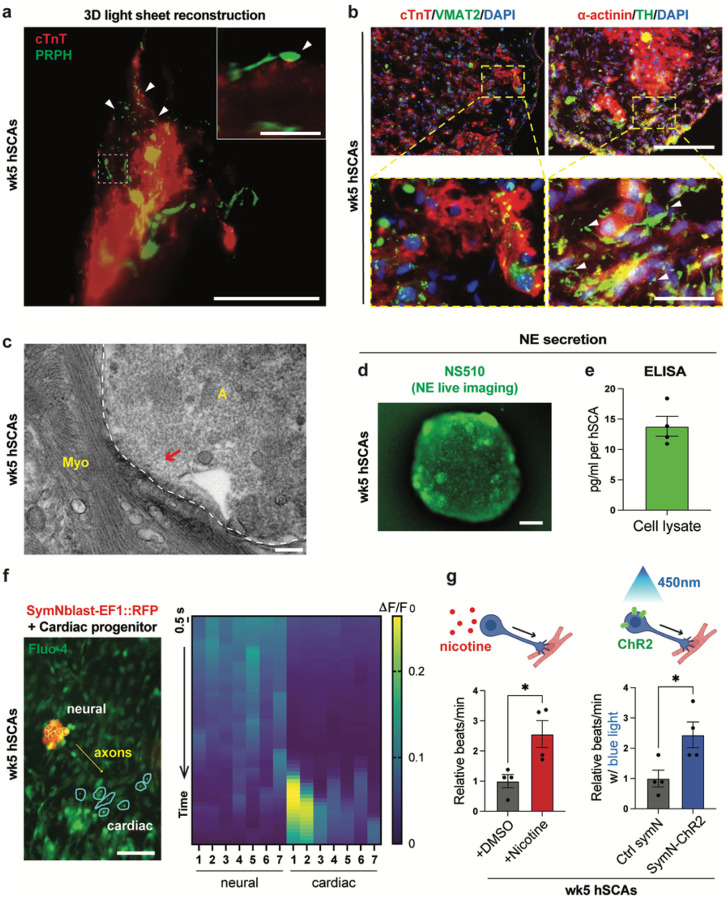
Sympathetic regulation in hSCAs. (**a**) Representative 3D reconstructive image of wk5 hSCAs using light sheet microscopy for cTnT and PRPH. Scale bars represent 100 μm, and 10 μm for the inset. White arrows indicate the nodal structure alone symN axons. (**b**) Representative cryosection image of wk5 hSCAs for symN axonal labeling using the combinations of cTnT/VMAT2 and TH/α-actinin. White arrows indicate the nodal structure alone symN axons. Scale bars represent 200 μm. (**c**) Representative TEM images showing the physical contact between symN axon (A) and heart muscles (Myo). White dashed line delineates the border of symN axonal terminal and muscle. Red arrow indicates the synaptic structure. Scale bar represents 200 nm. (**d**) Representative whole mount image of wk5 hSCAs using NS510. Scale bar represents 200 μm. (**e**) NE level in hSCA cell lysates was quantified by ELISA. n=4 biological replicates. (**f**) Left: Ca^2+^ imaging captured the functional coupling between symNs and cardiac tissues. Right: Quantification of the Ca^2+^ imaging recording showed the causal effect of symN activity to CM responsiveness. Scale bar represents 200 μm. (**g**) SymNs in wk5 hSCAs were activated by nicotine (NIC) or blue light. The changes of cardiac beating were quantified using image-based hSCA beating analysis. Unpaired Student’s t test. n=4 biological replicates. Error bars represent SEM. *, P<0.05.

Next, we asked whether the symNs in hSCAs are physically innervating the heart muscles. Using light sheet microscopy, we reconstructed the 3D muscle mass in hSCAs in high resolution (**movie S2**). Using whole mount staining, we observed that symNs were deeply associated with and growing throughout the cardiac tissues (Fig. 3a). The points of the physical contact between symN axons and cardiac tissues, which form classic swelling and nodal structures^45,46^, were also identified (Fig. 3a, arrows). We then stained the symN axon terminals with VMAT2 and TH and confirmed the co-localized signals of both within the cardiac tissue, as well as the nodal bouton en passant-like structure along the axon terminals (Fig. 3b, white arrows). Accordingly, TEM imaging also confirmed the physical innervation of symN axons to CMs in hSCAs (Fig. 3c).

Aside from the physical connection, we examined whether symNs in the assembloids are able to regulate cardiac function. NE is the main neurotransmitter used by symNs and is critical for functional regulation of the heart^20,47^. Thus, we sought to examine if symNs in hSCAs synthesize and release NE. NS510 is a highly sensitive NE chemical probe that has been used to study real-time NE synthesis and dynamics in chromaffin cells and symNs^29,48^. Using NS510, we detected NE throughout the assembloids (Fig. 3d). The NE level was also detectable in cell lysates by ELISA (Fig. 3e). To assess symN activity, we performed calcium (Ca^2+^) imaging using Fluo-4 Ca^2+^ labeling. To be able to distinguish Ca^2+^ fluxes in symNs and not in cardiac tissues, we differentiated symNblast using the EF1-RFP reporter hPSC line and mixed them with unlabeled cardiac progenitors (Fig. 3f). Over time, we observed Ca^2+^ sparks (green) first in symNs (red, Fig. 3f), followed by sparks in cardiac cells, which are downstream of the axons of the symNs (Fig. 3f). This result demonstrates that both symNs and cardiac tissues in hSCAs are functional, and cardiac activity can be regulated by symN activation. To test if we can manipulate hSCA beating by activating symNs, we used two methods to stimulate symNs within the hSCAs: (1) pharmacological activation using nicotine (1 μM), a widely used method in *in vitro* symN and CM cocultures that has been shown to exclusively activate symNs in short-term treatment^29,49–51^. (2) We differentiated symNblasts from an optogenetic iPSC line that expresses ChR2, and can be activated by blue light exposure^52^. Both nicotine treatment and blue light exposure resulted in increased hSCA beating efficiency (Fig. 3g). Together, our data suggest that symNs and cardiac tissues in hSCAs are functionally connected, and that the cardiac tissue can be manipulated by symN activation.

### SymNs regulate cardiac development through NE signaling

**Figure 4:**
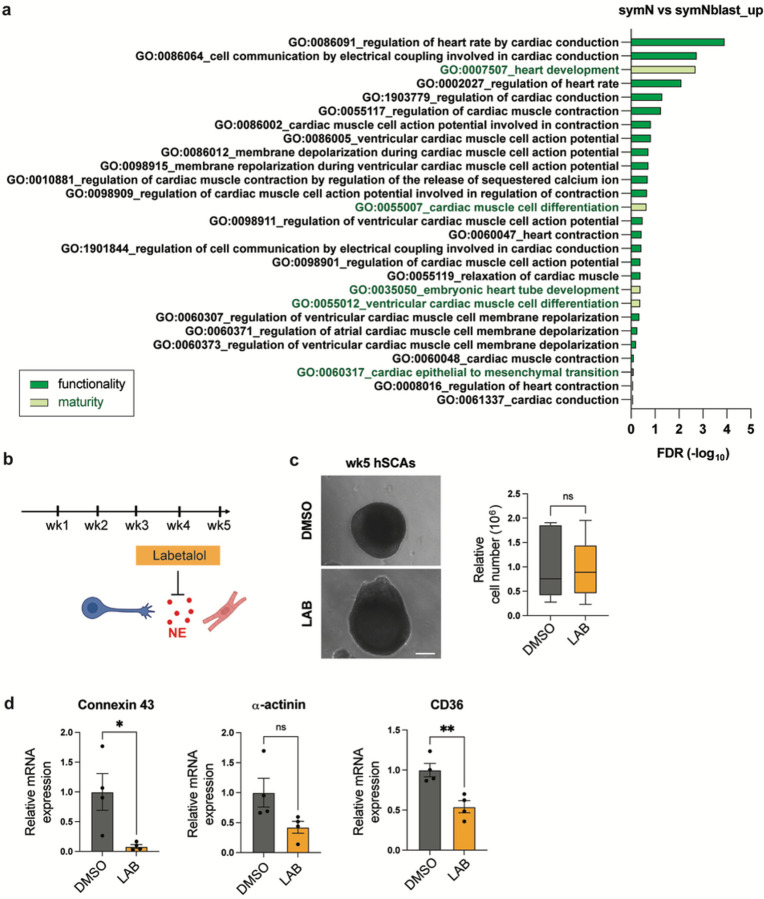
SymNs regulate cardiac development through NE signaling in hSCAs. (**a**) Selected upregulated GO terms that were involved in cardiac development and regulation during symN differentiation. (**b**) Schematic illustration of labetalol (LAB) treatment during hSCA development. (**c**) Left: Representative bright field image of wk5 hSCAs treated with LAB or DMSO. Right: Cell count of wk5 hSCAs treated with LAB or DMSO. Unpaired Student’s t test. n=6 biological replicates. Scale bar represents 200 μm. (**d**) RT-qPCR analysis of wk5 hSCAs with LAB or DMSO for cardiac maturity markers. Unpaired Student’s t test. n=4 biological replicates. Error bars represent SEM. *, P<0.05. **, P<0.01.

The effect of SymN activation on heart development has been reported, potentially through NE and adrenergic signaling^22,23^. *In vitro* 2D co-cultures using hPSC-derived or primary cultured symNs and CMs also demonstrated that symN connectivity facilitates CM maturation^53^. We re-analyzed bulk RNA seq. data comparing hPSC-derived symNBlasts (SSRN: https://dx.doi.org/10.2139/ssrn.4318816) and symNs^29^, and found that during maturation of the neurons alone (Fig. 1a), GO terms of genes that are involved in cardiac development and regulation pathways were significantly upregulated (Fig. 4a), suggesting that the neurons acquire the capability to regulate and mature cardiac tissue. To test if the symNs in our hSCAs play a regulatory role on cardiac development and maturation, we treated the assembloids with α- and β-adrenergic receptor antagonist labetalol (LAB, 1 μM) to fully block the NE signaling during the growth of the hSCAs (Fig. 4b). The drug was given from wk3, the stage when the assembloid growth almost reaches its plateau (Fig. 1e). Given that symN activity may promote cellular hyperplastic to hypertrophic transition of CM^22,23^, we first compared the overall size of hSCAs on wk5 after LAB treatment. We did not observe differences in assembloid size between DMSO or LAB treated hSCAs (Fig. 4c). However, RT-qPCR analysis for markers of CM maturity^53,54^ (*CX43, α-ACTININ*, and *CD36*) revealed decreased expressions upon LAB treatment (Fig. 4d). This result suggests that symNs promotes cardiac development through NE signaling in hSCAs; however, NE signaling alone may not be responsible for cardiac hypertrophic transition.

### hSCAs model hypoxia-induced cardiac infarction through endogenous NE

**Figure 5:**
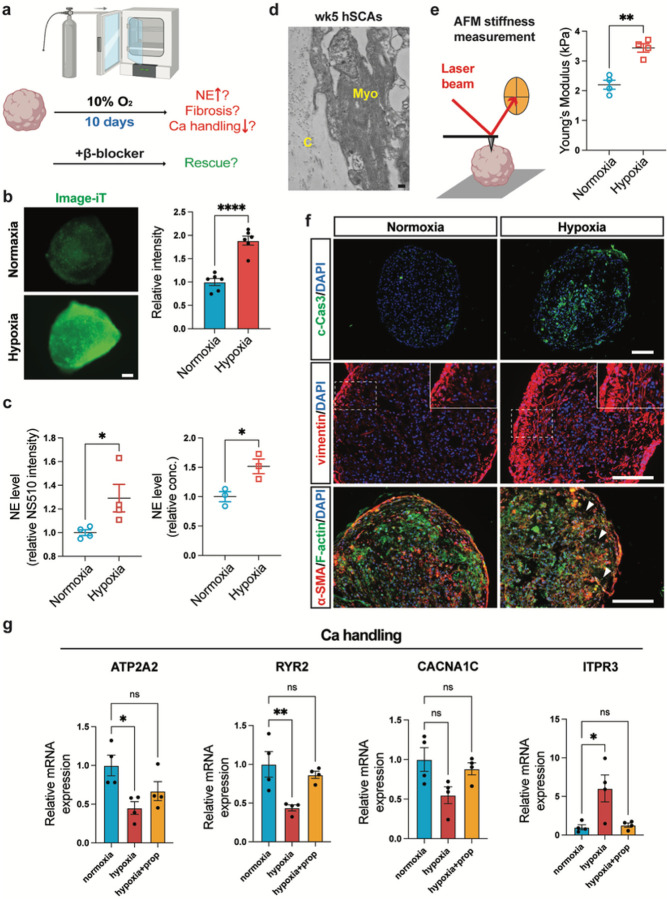
hSCAs model hypoxia-induced infarction. (**a**) Schematic illustration of hypoxia-induced cardiac infarction model on wk5 hSCAs. (**b**) Left: Representative whole mount images of wk5+10 days hSCAs labeled with Image-iT hypoxic dye. Right: Quantification of Image-iT intensity. Unpaired Student’s t test. n=6 biological replicates. Scale bar represents 200 μm. (**c**) NE levels of hSCAs in normoxic or hypoxic environments measured by NS510 staining (n=4 biological replicates) or ELISA (n=3 biological replicates). Unpaired Student’s t test. (**d**) Representative TEM images showing the extracellular matrix collagen (C) that is associated with the myofibers (myo). Scale bar represents 200 nm. (**e**) Left: Schematic illustration of stiffness measurement for hSCAs using AFM Right: AFM confirmed the increased stiffness of hypoxic hSCAs. Unpaired Student’s t test. n=4 biological replicates (**f**) Representative cryosection image of wk5+10 days hSCAs for cell death and fibrosis markers. Scale bars represent 200 μm. (**g**) RT-qPCR analysis of wk5+10 days hSCAs for calcium handling genes. Ordinary one-way ANOVA. n=4 biological replicates. Error bars represent SEM. *, P<0.05. **, P<0.01. ****, P<0.0001.

To test the effect of sympathetic input to cardiac function in the assembloids in a diseased state, we sought to model myocardial infarction. Richards et al. has established an elegant model of myocardial infarction in their cardiac organoids^55^. In this model, a moderate hypoxic environment (10% O_2_) was applied, which prevented massive and sudden cell death in 3D cardiac microtissues and allowed the observation of the progression of infarction. In addition to hypoxia, exogenous NE was added to the cardiac organoids to mimic the effects of increased sympathetic tone to the heart. The combined treatments resulted in infarction of the cardiac organoids at a state similar to mice with myocardial injury^55^. Here, we took advantage of this infraction model and applied their conditions to our hSCAs. We subjected hSCAs to 10% O_2_ for 10 days without exogenous NE and examined whether the symNs in the organoids become responsive to the hypoxic stress (Fig. 5a). After low oxygen treatment, hypoxic hSCAs were recognized by Image-iT^™^ Hypoxia Reagent compared to normoxic controls (Fig. 5b). The hypoxic stress stimulated overproduction of endogenous NE from symNs in hSCAs, measured via the NS510 probe and ELISA (Fig. 5c). Extracellular matrix in the heart, such as collagen, supports heart structural organization and development, but is also the component that forms the scar tissue in a damaged heart^56–58^. TEM imaging identified collagen in hSCAs (Fig. 5d). It has been shown that aberrant ECM accumulation and imbalanced degradation in the heart leads to the increased stiffness in cardiac fibrosis^56–58^. To evaluate this stiffening effect in our cardiac infarction mimicking hSCAs, we used atomic force microscopy (AFM) to measure the stiffness and compare it between normoxia or hypoxia-treated hSCAs (Fig. 5e and **Suppl. Figure 2**). As expected, hypoxic hSCAs showed increased stiffness compared to hSCAs in the normoxic environment (Fig. 5e). To further support the findings of fibrosis, hSCAs were stained for cleaved caspase-3 (c-Cas3) to detect apoptotic cells in the organoids. Compared to normoxic controls, hypoxic hSCAs displayed high amounts of c-Cas3^+^ cells in the center of the assembloids, likely due to the deficiency in oxygen supply (Fig. 5f). In addition, using the cardiac fibroblast marker vimentin that labels fibrotic tissue, we observed increased vimentin signals on the outskirt of the hypoxic hSCAs. Vimentin^+^ cells in that area showed elongated and elastic morphology, which is a typical feature of fibrotic tissues^55^ (Fig. 5f). We further confirmed the fibrotic cardiac scar tissues in hypoxic hSCAs by assessing the colocalizing level of α-SMA and F-actin^55^. In the outskirt area, hypoxic hSCAs showed higher colocalization of α-SMA^+^/F-actin^+^ cells compared to normoxic hSCAs (Fig. 5f). RT-qPCR analysis showed that the expression of Ca^2+^ handling genes^55^ were altered in hypoxic hSCAs, with a pattern similar to what Richards et al. showed, suggesting that Ca^2+^ handling capacity was impaired in hypoxic hSCAs (Fig. 5g). hSCAs present a powerful tool to screen drugs for heart failure. In the clinic, β-blockers (propranolol, a βAR antagonist), which blocks the excitatory effects of NE to the heart are prescribed for heart failure and to prevent a second infarction^59,60^. Thus, to further evaluate the potential of hSCAs for future cardiotoxicity studies, we treated hypoxic hSCAs with propranolol (Fig. 5a). When treating hSCAs with propranolol (1 μM) in addition to low oxygen, the impaired expression of Ca^2+^ handling genes were rescued (Fig. 5f). These results suggest that hSCAs possess a functional symN-cardiac tissue axis, which is responsive to environmental inputs at both healthy and diseased states and may be used for cardiotoxicity screening.

## Discussion

Organoids are self-organizing 3D cell cultures that mimic some of the cellular, structural, and functional complexity of the native organ *in vitro*. They provide valuable insights to understand the structure-function relationship of human organs that 2D models cannot achieve, such as brain lobe structure, renal pyramid function and heart spatial patterning. Assembloids result from the integration of multiple organoids or combination of organoids with other cell types^1–3^. They have the advantage of enabling the study of interaction of tissues that may not normally develop from the same progenitor, for example forebrain and hindbrain^1^ or vasculature and brain tissue^61^. A major outstanding issue in the organoid/assembloid field is the lack of innervation of peripheral tissue organoids. This is despite the fact, that almost all organs outside the brain are innervated by the peripheral nervous system, and that this neural regulation is crucial to the development, integrity, and function of organs.

Few innervated organoids have been reported to date; however, the common disadvantage of those exciting reports is that they cannot be easily adapted to other organ type organoids or are technically difficult to reproduce by researchers. Workman et al. mixed specified hPSC-derived vagal NC cells with developing gut tube organoids and created an intestinal organoid with enteric neuron innervation. This elegant work laid the conceptual groundwork for innervating peripheral tissue organoids^9^. However, enteric neurons are specific to the GI tract and do not innervate other organs. Thus, making this less universal as an innervation strategy for many different organoid types. Schneider et al. created a 3D bioengineered hPSC-derived CM model with autonomic innervation using hPSC-derived autonomic organoids^12^. CMs in this model were plated in circular form on dynamic stretch devices, which achieved advanced maturation of cardiac tissues. Autonomic neural organoids were then stuck into the ring of the engineered cardiac organoids for innervation and regulation thereof. However, these assembloids did not mimic the heart structures (ventricle/atrial patterning, heart cavity, for instance) and required specific instrumentation for their analysis. Innervated muscle models, where motor neurons connect to skeletal muscle, have been achieved using the one-pot differentiation strategy from the neuromesodermal progenitors^10^. This demonstrated the possibility to study complicated and anatomically distal functional coupling in organoids. However, not many organs develop their cell types and innervation from a common progenitor, such as the neuromesodermal progenitor, thus this strategy is not easily transferrable to other organoids. The assembloid technology, on the other hand, presents an ideal modular platform for individual organoid components, that is suitable for innervation of organoids^2,3^.

Here, we describe a strategy to address the need of a method to innervate any organ type organoid relatively easily with symNs. As an example, we created cardiac organoids that are innervated by symNs. We purposely used a simple, easy to reproduce, and relatively low-cost assembly method, that does not require bioengineering or special instrumentation, with the goal for many researchers to be able to reproduce this technique. We created the assembloids in a modular way, so that researchers can adapt them to their needs. For example, one could use the symNs to innervate other organoids, such as lung, kidney, or liver. Or one could replace the symNs and innervate the cardiac organoids with parasympathetic neurons (SSRN: https://dx.doi.org/10.2139/ssrn.4318816) or sensory neurons^62,63^.

Organoids or assembloids must fulfil certain criteria to be useful^3,28,40^. (1) 3D cultures. Several groups have generated 2D co-culture models of symN-innervated CMs in recent years. In 2016, Oh et al. demonstrated a functional coupling 2D co-culture model, using hPSC-derived symNs and mouse neonatal ventricular myocytes, and showed that the beating rate of CMs can be regulated by symNs, and can be manipulated with nicotine or optogenetic stimulation^50^. Later, Larsen et al. established a 2D co-culture system of neonatal ventricular myocytes and sympathetic stellate neurons from control Wistar Kyoto (WKY) and pro-hypertensive (SHR) rats. They found that hypertensive symNs were able to induce hypertensive phenotypes in healthy CMs, while healthy symNs rescued the hypertensive state in CMs^51^. In 2020, Winbo et al. performed the 2D co-culture model using symNs and CMs both derived from hPSC, which also displayed functional coupling regulation of CMs through symNs, inducible by nicotine^49^. In 2022, we described that symNs derived from iPSCs from the genetic autonomic disorder Familial Dysautonomia (FD) were hyperactive and in 2D co-cultures increased hPSC-derived CM beating^29^. Adding the option of a 3D organoid model to this toolset for disease modeling will increase its power for discovery of disease mechanisms and drug discovery. Our hSCAs are cultured in 3D throughout their generation and maturation stages (Fig. 1). (2) Self-organization from stem or progenitor cells. Our hSCAs are assembled by mixing day 14 symNblasts and day 7 cardiac progenitors (Fig. 1). We^29,30^ and others^35,36^ have previously shown that these progenitors will form fully differentiated and functional symNs or cardiomyocytes upon continued 2D culture. Furthermore, these symNs have been employed by us to modeled autonomic dysfunctions in Familial Dysautonomia, within the SARS-CoV-2 infection milieu, and under diabetic hyperglycemia conditions^29,64,65^. (3) Contain multiple cell types that mimic the native organ. We show here that the hSCAs contain multiple cardiac and symN lineage cell types. The human heart consists of multiple cardiac lineages in addition to CMs, such as endothelial cells, smooth muscle cells, and cardiac fibroblast^33,34^. Our hSCAs contained mature cardiac muscle fibers, T-tubules, cardiac fibroblasts, epicardial and endocardial layers, as well as atrial and ventricular CMs (Fig. 2b–d and h–i, and 5f). 4. Mimicking some structural and functional features of the native organ. hSCAs contained CM-innervating functional symNs, in which NE synthesis was detected (Fig. 3a–e). The functional coupling between symNs and CMs was observed by Ca^2+^ imaging and could be manipulated by nicotine and optogenetic stimulation (Fig. 3f–g).

It is becoming clearer that tissue innervation is essential for proper development, maturation and even repair of most organ tissues^66–71^. Accordingly, symN innervation of the heart plays an important role in development and reciprocal maturation of the tissues. In 2015, Kreipke et al. used the neurotoxin 6-hydroxydopamine (6-OHDA) to induce symN lesions in neonatal mouse hearts and found that the proliferation of CMs was increased, indicating disrupted cell cycle withdrawal due to the lack of symN innervation^22^. In 2021, Tampakakis et al. demonstrated similar results using a smooth muscle-specific NGF deprivation mouse model, which resulted in heart-specific symN depletion in embryonic hearts, as well as increased CM proliferation^23^. Interestingly, while both studies showed increased proliferation of CMs due to the absence of symN innervation, the model by Kreipke et al. showed decreased heart size after symN depletion, whereas Tampakakis et al. showed enlarged heart size. This might be due to the difference in CM density (unchanged in Kreipke et al. and increased in Tampakakis et al.) in each model and the unknown effect of symN signaling to other cell types that form the heart mass^22,23^. Additionally, it is believed that such regulation on developmental cardiac hypertrophy by symNs is mediated by NE and adrenergic signaling^72,73^. Indeed, in 2022, Kowalski et al. co-cultured mouse primary symNs with hPSC-derived CMs and showed that with symN innervation, the mature cardiac gene expression and functional cardiac activity were improved^53^. However, they also found that treating hPSC-derived CMs alone with isoproterenol, a β-adrenergic receptor agonist, was not sufficient to induce such maturity improvement without physical connection with symNs^53^, implying undiscovered mechanisms in the symN-heart axis, which may also account for the reason of different heart sizes observed in the models above (P0 symN depletion in Kreipke et al., in which embryonic innervation remained, versus symN null in the heart in Tampakakis et al.). The hSCAs described here, therefore, might be an ideal model to assess development and reciprocal maturation of symNs and CMs in cultures. Using hSCAs, we modeled early heart development. Since the exact effect of NE on CMs is not fully clarified^22,23,53^, we used α- and β-adrenergic receptor antagonist LAB to fully block the entire downstream target of NE (Fig. 4b). In our model, hSCA size and cell number were not altered in LAB-treated organoids compared to control (Fig. 4c). Additionally, genes for cardiac maturation decreased upon LAB treatment, indicating the importance of functional symN signaling for heart development and maturation. Future studies using hSCAs may focus on identifying the effect of other symN cofactors, such as neuropeptide Y on cardiac maturation, as well as the levels of cardiac maturation in different cardiac compartments using single cell RNA sequencing.

Finally, organoid technology has become an important tool for disease modeling approaches^4–8,31,32,44,74^. In line with such studies, we employed our hSCAs to model the hypoxia-induced cardiac infraction (Fig. 5a). We successfully recapitulated the endogenous NE crisis in hSCAs from symNs upon hypoxic stress, which caused cardiac fibrosis that was rescued by treatment with the β blocker propranolol along with the hypoxic stress (Fig. 5b–g). There are a few limitations in this model. First, in the whole organism, other NE releasing tissues, such as adrenal chromaffin cells, can also contribute to the NE crisis. Second, as a critical part during heart failure, the inflammation response was not recapitulated in hSCAs, due to the lack of immune cells in the organoids. Such challenges may be addressed in the future by incorporating more distal tissues, such as chromaffin or immune cells into a further advanced assembloid.

## Online methods

### hPSC maintenance

Human embryonic stem cell (hESC) line WA09 (H9) and optogenetic iPSC line hiPSC(ChR2/NpHR)^52^ were mainly used in this study. hPSCs were maintained in Essential 8 medium (Gibco, A15170–01) on vitronectin coated (Thermo Fisher/Life Technologies, A14700, 5 μg/ml) cell culture plates, and passaged using EDTA (Sigma, ED2SS) as previous descrobed^30^.

### hSCA differentiation

#### SymNblast.

A detailed differentiation protocol can be found in our previous publication^30^. Day 0: hPSCs were dissociated by EDTA and replated on Geltrex (Invitrogen, A1413202)-coated plates at 125×10^3^ cells/cm^2^. Cells were fed with day 0–1 medium: Essential 6 medium (Gibco, A15165–01), 0.4 ng/ml BMP4 (PeproTech, 314-BP), 10 μM SB431542 (R&D Systems, 1614) and 300 nM CHIR99021 (R&D Systems, 4423). From day 2 on, cells were fed with day 2–10 medium: Essential 6 medium, 10 μM SB431542 and 0.75 μM CHIR99021. For the best result, we strongly recommend to perform BMP4 titrations for each batch/lot due to the batch-to-batch variability of BMP4. NC cell fate should be induced by day 10. Day 10 NCCs were dissociated by accutase (Coring, AT104500) and replated to ultra-low attachment plates to form symNblast spheroids. The symNblast medium for day 10–14 contains Neurobasal medium (Gibco, 21103–049), B27 (Gibco, 17502–048), N2 supplement (Thermo Fisher/Gibco, 17502048). L-Glutamine (Thermo Fisher/Gibco, 25030–081), 3 μM CHIR99021 and 10 ng/ml FGF2 (R&D Systems, 233-FB/CF).

#### CM progenitor.

A detailed differentiation protocol is described by Lin et al.^36^. On day − 1, hPSCs were replated by EDTA at a Matrigel (Corning, 1:20)-coated plates at 250×10^3^ cells/cm^2^. Next day on (day 0), medium was changed to CDBM base medium: DMEM/F12 (Gibco, 11320033), 64 mg/L ascorbic acid (Sigma, A8960), 13.6 μg/L sodium selenium (Sigma, S5261), 10μg/ml transferrin (Sigma, T3309) and Chemically Defined Lipid Concentrate (Gibco, 11905031). On day 0, 5 μM CHIR99021 was added to CDBM medium. On day 1/5/6, 0.6 U/ml heparin (STEMCELL Technologies, 07980) was added to CDBM medium. On day 2/3/4, 0.6 U/ml heparin and 3 μM XAV were added to CDBM medium.

hSCA assembly. Day 14 symNbalsts and day 7 cardiac progenitors were dissociated by accutase. Cells were fully mix at 1:1 ratio as 100×10^3^ symNblasts + 100×10^3^ cardiac progenitors on 96-well ultra-low attachment plates to form one organoid per well, or as 500×10^3^ symNblasts + 500×10^3^ cardiac progenitors on 24-well ultra-low attachment plates for bulk organoid generation (about 5 organoids will form per well). From now on, assembloids were fed with hSCA medium: Neurobasal medium + CDBM base at 1:1 ratio, 100x B27, 200x L-Glutamine, 200x N2, 12.5 ng/ml GDNF, 12.5 ng/ml BDNF, 12.5 ng/ml NGF, 100 μM ascorbic acid, 100 μM dbcAMP, 0.0625 μM retinoic acid (add RA freshly every feeding), and 10 μg/ml insulin (Sigma, I-034). hSCAs were fed every 3 days.

### RT-qPCR

0.5×10^6^ cells were collected using Trizol (Invitrogen, 15596026) for each sample. Reverse transcription with 1 μg total RNA was performed using iScript^™^ Reverse Transcription Supermix (Bio-Rad, 170884). SYBR green (Bio-Rad) RT-qPCR was ran using CFX96 Touch Deep Well Real-Time PCR Detection System (Bio-Rad), and analyzed by CFX Maestro. Primers used in this study are listed in **supplemental Table 1.**

### Image based beat quantification

hSCA beating was analyzed using the ImageJ Time Series Analyzer plugin (Balaji J. UCLA). hSCA beating video was saced as AVI file for the analysis.

### Multielectrode array (MEA)

To measure cardiac signaling, hSCAs were placed on dry MEA plates (Axion BioSystems, BioCircuit or CytoView), one organoid per 96 well. After sitting for a few minutes, hSCA medium was added on top of the slightly attached organoids in 20–50 μl droplets. Do not fully cover the entire bottom area of the well to prevent the organoids from floating. hSCA field potential was measured using a MEA plate reader (Axion BioSystems, Maestro Pro) under the cardiac detection mode according to manufacturer’s instruction.

### Cryo-sectioning

hSCAs were fixed by 4% paraformaldehyde overnight and washed twice by PBS. Fixed hSCAs were dehydrated by 30% sucrose in PBS overnight until the organoids sink to the bottom. hSCAs were then embedded in OCT compound (Sakura Finetek) and cryo-sectioned at 7 μm thickness.

### Immunohistochemistry

#### Cryo-section staining.

Sections were permeabilized and blocked by 0.2% Triton X-100 and 3% goat or donkey serum in PBS for 60 minutes, and incubated with primary antibodies in the blocking buffer overnight at 4°C. Sections were rinsed twice by PBS incubated with secondary antibodies in the blocking buffer overnight at room temperature (RT). After PBS wash, sections were mounted using mounting medium with DAPI (Abcam, ab104139).

#### Whole mount staining.

Fixed hSCAs were permeabilized by 0.3% Triton, 1% BSA and 3% goat or donkey serum in PBS for 2–4 hours at RT. Primary antibodies were added and incubated for 48 hours at 4°C. After PBS wash for one hour at RT, secondary antibodies were added and incubated for 24 hours at 4°C. After PBS wash for one hour at RT, DAPI was added and incubated for one hour at RT.

Fluorescent images were taken using the Lionheart FX Automated Microscope. Primary and secondary antibodies used in this study were listed in **supplemental Table 2.**

### Wheat germ agglutinin (WGA) staining

hSCA cryo-sections were incubated with 5 μg/ml 488-conjugated WGA (biotium, 29022) for 30 minutes at RT. Permeabilization was performed afterward and regular immunostaining was performed after WGA staining.

### Phalloidin staining (for F-actin)

Phalloidin-iFluor 488 Reagent (Abcam, ab176753, 1:1000) was used according to manufacturer’s instructions. Cryo-sectioned hSCAs were permeabilized and blocked by 0.2% Triton X-100 and 3% goat or donkey serum in PBS for 60 minutes, then incubated with 1x Phalloidin solution in PBS with 1% BSA for 30 minutes. Sections were washed by PBS for at least 3 times. General immunohistochemistry staining can be performed after Phalloidin staining.

### Norepinephrine live labeling

The NE tracer (NS510) was a kind gift by Timothy Glass’s laboratory at University of Missouri. hSCAs were incubated with 1 μM NS510 in hSCA culture medium and at 37°C for 60 min. After PBS wash for at least twice, hSCAs were imaged using Lionheart FX Automated Microscope at 440 nm excitation and 520 nm emission.

### NE ELISA

NE assay was performed according to manufacturer’s instructions (EagleBio, NOU39-K01). hSCA lysates from 1 well of 24-well plate were collected in 200 μl PBS. To preserve NE, sample stabilizer included in the kit was added to each sample. Lysate solutions were spun at 300 × g for 5 min to remove debris. The samples were ready for NE detection or were stored at − 80°C for long-term storage (although not recommended).

### Light sheet microscopy

The imaging of the hSCAs was conducted using a custom-built light-sheet microscope^75^. The microscope was outfitted with a 16x/0.8 NA water-immersion detection objective (Nikon N16XLWD-PF). For the excitation of GFP fluorescence, a 488 nm laser was employed, operating at a power density of 1.86 W/cm^2^. Similarly, a 561 nm laser was used to stimulate RFP fluorescence, also at a power density of 1.86 W/cm^2^. The light sheet’s thickness at the beam waist was 6.7 μm. An exposure time of 50 ms was maintained during imaging. The volumetric images captured were 100 × 276.48 × 276.48 μm3 in size (200 × 2048 × 2048 pixels), offering a resolution of 1.64 × 0.313 × 0.313 μm. For sample preparation, hSCAs were immobilized in a solution of 1% low melting point agarose (Sigma) with 1% PBS (Gibco), subsequently placed within a petri dish for stable imaging.

### Transmission electron microscopes (TEM)

hSCAs were fixed in Trump’s EM fixative: [4% paraformaldehyde, 1% glutaraldehyde in 0.1M Phosphate buffer, pH 7.25] and washed several times in 0.1M Phosphate buffer before post-fixation in 1% osmium tetroxide in buffer for 1 hour. The organoid samples were washed several times in deionized water and then placed in a 0.5% aqueous uranyl acetate enbloc for 1 hour in the dark. After several more washes in deionized water, the organoid samples were dehydrated in an ethanol series [30%, 50%, 75%, 95%, 100%], cleared in two changes of acetone, and two changes of propylene oxide. The organoid samples were infiltrated with 2:1, 1:1 and 1:2 mixtures of propylene oxide and Mollenhauer’s Epon-Araldite plastic mixture^76^ two hours respectively; then two changes of 100% Epon-Araldite plastic for at two hours each before embedding the tissues in flat embedding molds. The embedded samples were polymerized in a 70–80° C oven overnight^77^. 1μm sections from the polymerized blocks were obtained using a Reichert Ultracut S ultramicrotome. Sections were placed on glass slides and stained with 1% Toluidine Blue O in 1% sodium borate. The stained sections were evaluated, and areas of interest were chosen before trimming the corresponding block face for thin sectioning. 60–70nm sections were obtained and placed on 200-mesh copper Locator grids. One of the grids was post stained with 2% aqueous uranyl acetate and Reynolds lead citrate (Reynolds, 1963), while the remaining grids were left unstained. Grids were viewed with a JEOL JEM-1011 transmission electron microscope at varying magnifications using an accelerating voltage of 100 KeV. Images were acquired using an AMT XR80M Wide-Angle Multi-Discipline Mid-Mount CCD Digital Camera with a resolution of 3296 × 2460 pixels.

### Calcium imaging

hSCAs were incubated with 4 μM of Fluo-4 AM (TOCRIS, 6255) in hSCA culture medium and incubated at 37°C for 30 minutes. After PBS wash for three times, hSCAs were incubated for 30 min with fresh medium at 37°C. After the incubation, hSCAs were read and video was made using Lionheart FX Automated Microscope.

### Optogenetic stimulation

hSCAs that are consist of hiPSC(ChR2/NpHR)-derived symNs were stimulate by blue light (HQRP) using the High-Accuracy Digital Electronic Timer (GraLab, model 451) in 1 second on, 4 second off frequency for 5 minutes.

### Low oxygen model for cardiac infarction

Wk5 hSCAs were fed with hSCA medium and placed into the hypoxia chamber (incubator subchamber system with O_2_/CO_2_ setpoint control, BioSpherix) with 10% O_2_, control group was maintained in regular culture condition (21% O_2_). hSCAs in both conditions were fed with fresh hSCA medium every 3 days for 10 days.

### Atomic force microscopy (AFM)

hSCAs were fixed by 4% PFA for 24 hours before AFM measurement. Stiffness was measured by AFM (Agilent Technologies 5500 Scanning Probe Microscope) using Aluminum coated cantilever (ASPIRE CCSR-10, spring constant = 0.1N/m). Fixed hSCAs were placed on the surface with minimal liquid remaining, and measured immediately. The indentation measurement up to the depth about 200 μm. The force-distance curves collected were fitted with the modified Hertz model to calculate the Young’s Modulus.

### Image-iT^™^ hypoxia staining

4 μm Image-iT^™^ Green Hypoxia Reagent (ThermoFisher, I14833) was given to hSCAs and incubated at 37°C for 60 minutes. Stained hSCAs were washed by PBS for 3 time and imaged by Lionheart FX Automated Microscope.
